# Correlation between surface texture and internal defects in laser powder-bed fusion additive manufacturing

**DOI:** 10.1038/s41598-021-02240-z

**Published:** 2021-11-24

**Authors:** Makiko Yonehara, Chika Kato, Toshi-Taka Ikeshoji, Koki Takeshita, Hideki Kyogoku

**Affiliations:** 1 Fundamental Technology for Next Generation Research Institute, Kindai University, 1 Takaya-Umenobe, Higashi-Hiroshima, Hiroshima, 739-2116 Japan; 2 Technology Research Association for Future Additive Manufacturing, Kindai University Hiroshima Branch, 1 Takaya-Umenobe, Higashi-Hiroshima, Hiroshima, 739-2116 Japan; 3 Nikon Corporation, 6-3, Nishioi 1-chome, Shinagawa-ku, Tokyo, 140-8601 Japan

**Keywords:** Engineering, Materials science

## Abstract

The availability of an in-situ monitoring and feedback control system during the implementation of metal additive manufacturing technology ensures that high-quality finished parts are manufactured. This study aims to investigate the correlation between the surface texture and internal defects or density of laser-beam powder-bed fusion (LB-PBF) parts. In this study, 120 cubic specimens were fabricated via application of the LB-PBF process to the IN 718 Ni alloy powder. The density and 35 areal surface-texture parameters of manufactured specimens were determined based on the ISO 25,178–2 standard. Using a statistical method, a strong correlation was observed between the areal surface-texture parameters and density or internal defects within specimens. In particular, the areal surface-texture parameters of reduced dale height, core height, root-mean-square height, and root-mean-square gradient demonstrate a strong correlation with specimen density. Therefore, in-situ monitoring of these areal surface-texture parameters can facilitate their use as control variables in the feedback system.

## Introduction

Metal additive manufacturing (AM), especially powder-bed fusion (PBF), is considered an important process in the creation of new materials featuring exquisite material properties and complex structures. This process involves the use of a laser or an electron beam as the heat source^1–3^. However, several concerns have been raised regarding the quality of finished parts obtained using the PBF process. These concerns relate to the occurrence of such defects as pores and lack of fusion in the finished parts as well as the deterioration in surface roughness during processing owing to characteristics intrinsic to the PBF process^4,5^. Accordingly, the mechanical properties and surface roughness of PBF-manufactured parts are found to be inferior than those of wrought materials^1,2^. Thus, the demand to minimize the occurrence of defects during PBF processing and ensure high quality of finished parts has led to the development of monitoring and feedback control systems^4,5^.

For the development of the said system, it is important to elucidate the physical phenomena that occur during the laser-beam PBF (LB-PBF) process. Powder characteristics affect their recoating behavior, thereby influencing the melting and solidification phenomena caused by laser irradiation, which in turn, affect the quality of finished parts^6–9^. Extant studies have investigated the melt-pool behavior as well as the cause of the occurrence of defects, such as pores and lack of fusion, through not only experiments involving the use of a test bench and high-speed camera but also numerical simulations^10–18^. Efforts have been made to control the laser-radiation level by monitoring the melt-pool state parameters, such as its configuration and temperature^10^. Other prior studies^19–22^ have investigated means to identify the melting and solidification phenomena by performing experiments involving the use of a test bench equipped with a 1-kW high-power single-mode fiber laser, high-speed camera, and thermo-viewer. Numerical simulations have also been performed in these extant studies. Moreover, Calta et al.^23^ and Martin et al.^24^ have investigated the keyhole- and pore-formation phenomena using in-situ time-resolved X-ray imaging and diffraction analysis. Additionally, Zhao et al.^25^ and Cunningham et al.^26,27^ investigated keyhole formation and spattering using the ultra-high-speed synchrotron x-ray imaging.

The demands pertaining to the quality of the finished parts include meeting standardized mechanical-property requirements, surface-roughness criteria, and accuracy. These properties are influenced by the melting and solidification phenomena caused by laser radiation. Thus, the occurrence of internal defects and inconsistencies in surface roughness during melting and solidification are considered to influence the quality of finished parts. The influence of process parameters on the occurrence of internal defects and/or density of finished parts has been frequently reported^22^. Accordingly, the laser power, scan speed, hatching pitch, and layer thickness have been identified as essential LB-PBF-LB process parameters. The laser-power and scan-speed process maps are used determine optimum process parameters^21,28^. Aoyagi et al.^29^ employed support vector machines to construct a process map for the electron-beam PBF (EPBF) process. Gobert et al.^30^ employed supervised machine learning for defect detection during the PBF process. In addition, the volumetric energy density (energy density) is widely adopted as a process parameter owing to its strong correlation with the defects in^23^ and density of finished parts. The influence of process parameters on the surface roughness of parts fabricated via LB-PBF is reported in^31^. Although the relationship between process parameters and surface roughness obtained via line measurement has been reported in^32–34^, Whip et al.^35^ have reported that the application of the ISO 25,178–2 areal surface-texture parameters^36^ is adequate to describe the surface roughness of AM parts. They investigated the relation between process parameters (laser power and scan speed) and seven different standard height-based surface-roughness metrics^35^. Of these, the arithmetic mean height (*S*_a_), maximum valley depth (*S*_v_), reduced dale height (*S*_mr2_), and reduced valley depth (*S*_vk_) were observed to decrease with an increase in laser power. Meanwhile, different trends were observed corresponding to changes in the scan speed. Moreover, Thompson et al.^37^ and Gomez et al.^38^ have investigated methods to determine the adequate surface texture of AM parts. As reported by Gomez et al.^38^, the coherence scanning interferometry (CSI) technique yields excellent surface-topography measurement results for metal AM surfaces with varying roughness levels and slope distributions. Gockel et al.^39^ concluded that *S*v demonstrates a much stronger correlation with fatigue life than *S*a. Eidt et al.^40^ investigated the influence of process parameters on the surface texture of the down skin and reported that the surface roughness deteriorates with an increase in laser power. Taylor et al.^41^ investigated the correlation between the surface-texture parameter, energy density, and material density, and they reported the root-mean-square slope (*S*Δq) as a better delineator of different LB-PBF parts than *S*a. Fleming et al.^42^ proposed a manual closed-loop control system with in-situ coherent imaging to prevent the occurrence of defects. This system facilitated the determination of the surface-texture parameters, recoater-blade damage, and powder-packing density via in-situ monitoring of the layer-thickness deviation and *S*_a_. Thus, the correlation between the surface-texture parameters and density or internal defects is yet to be quantitatively investigated in a systematic manner. Therefore, the accurate prediction of internal defects using the surface-texture parameters remains a challenging task, as reported in^9^.

This study aims to investigate the correlation between the surface texture and internal defects or density of LB-PBF parts, thereby providing guidelines for the development of an in-situ monitoring and feedback control system capable of preventing defect occurrence in LB-PBF parts.

## Results and discussion

### Relationship between process parameters and density

**Figure 1:**
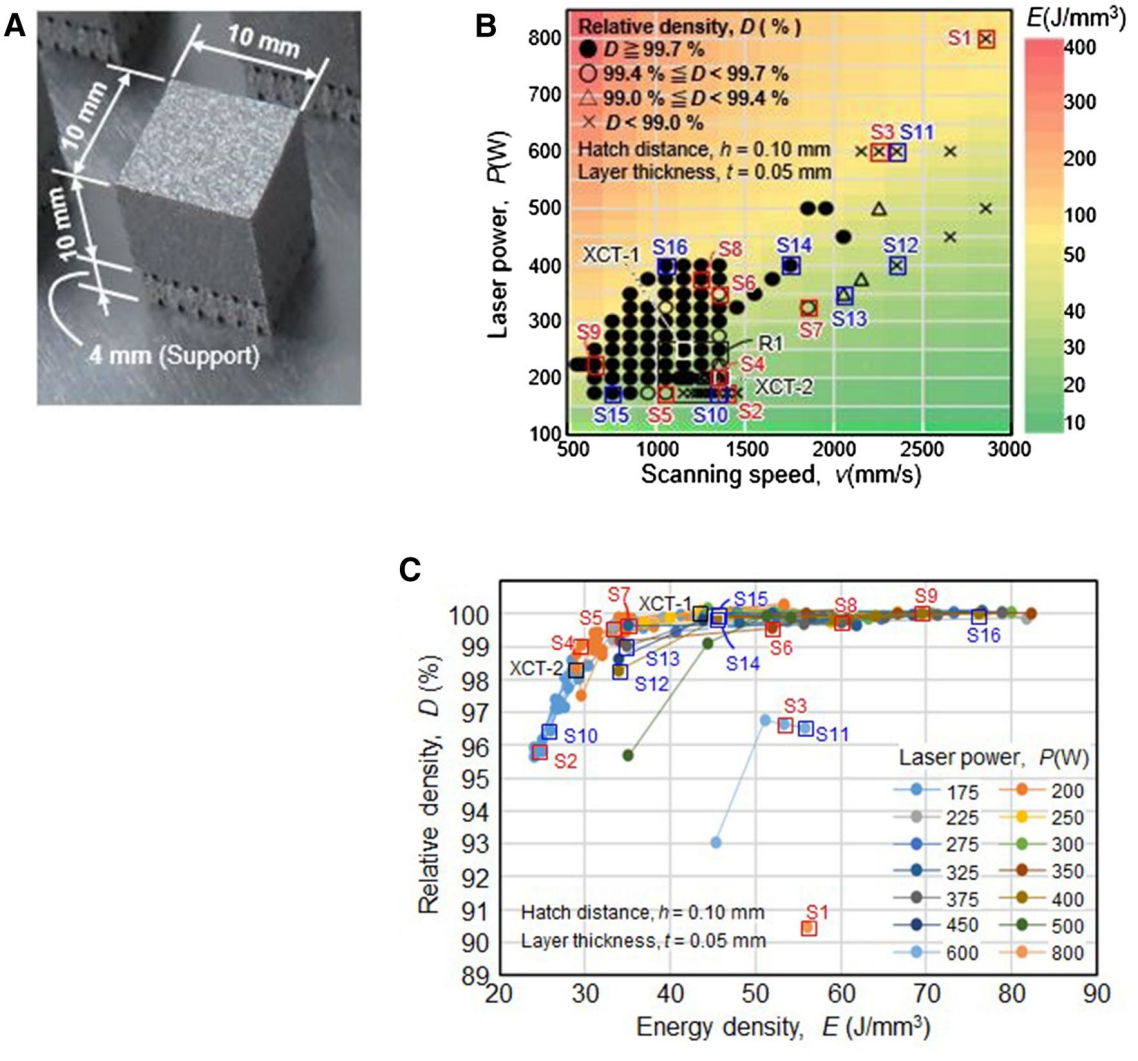
Relationship evaluated by the relative density between process parameters and density, (**A**) cubic specimen; (**B**) process map between laser power and scan speed; (**C**) relationship between relative density and energy density. Images created using Microsoft Office Excel 2019 (https://www.microsoft.com/ja-jp/microsoft-365/excel).

Experiments performed in this study involved the fabrication of cubic specimens of Inconel 718 (IN 718) measuring 10 × 10 × 10 mm (Fig. 1A). A PBF test bench equipped with a 1-kW single-mode fiber laser was used for specimen fabrication, which was performed in a nitrogen environment (oxygen content < 0.1 wt%). Other operating conditions considered during fabrication included laser spot diameter *d* = 100 µm (1/e^2^), laser power *P* = 175–800 W, scan speed *v* = 550–2850 mm/s, hatching pitch *h* = 0.10 mm, layer thickness *t* = 0.05 mm, and energy density *E* = 24.1–82.4 J/mm^3^. The energy density *E* was calculated using the relation $E=\frac{P}{vht}$. Thus, the relationship between process parameters and density was investigated over a wide range of laser power (up to 800 W) and scan speed (up to 2850 mm/s) values, which greatly exceeded those reported in extant studies^18–22^.

The process map of laser power versus scan speed evaluated using the relative-density values obtained for 120 cubic specimens fabricated in this study is depicted in Fig. 1B. The relative density was calculated by dividing the Archimedes-principle-based density of as-built specimens by their corresponding true density (8.20 g/cm^3^). The region of low power and low scan speed in Fig. 1B corresponds to high relative-density values that exceed 99.7%. Meanwhile, the region characterized by high power (exceeding 400 W) and high scan speed (exceeding 2000 mm/s) is representative of low relative densities. As reported in a prior study^20^, the observed reduction in density can be attributed to the lack of fusion caused by the occurrence of melt-pool instability and intense spattering. In addition, the use of high laser power results in the generation of keyhole pores. The occurrence of the melting and solidification phenomena under high laser power and high scan speeds is seldom reported in prior investigations except^22^. On the contrary, the reduction in specimen density is a common observation indicating consideration of unfavorable fabrication conditions, including use of low power and low scan speed.

The relationship between the energy density and relative density of the fabricated cubic specimens is depicted in Fig. 1C. The relative density increases with energy density and attains its maximum value (referred to as "full density") corresponding to an energy–density value of approximately 35 J/mm^3^. A similar trend can be observed for other materials, albeit it depends on not only the material but also the powder and laser characteristics. Although most cases, including that corresponding to 400-W laser power, follow this trend, few cases do not comply with the same. These deviating cases correspond to those involving use of high laser power (exceeding 400 W) and high scan speed (2,000 mm/s). The discrepancy in collected data can be attributed to the occurrence of intense spattering due to melt-pool instability, as previously reported^22–24^, as well as the generation of residual keyhole pores at high power and scan speed, as reported by Zhao et al.^25^ and Cunningham et al.^26,27^.

**Figure 2:**
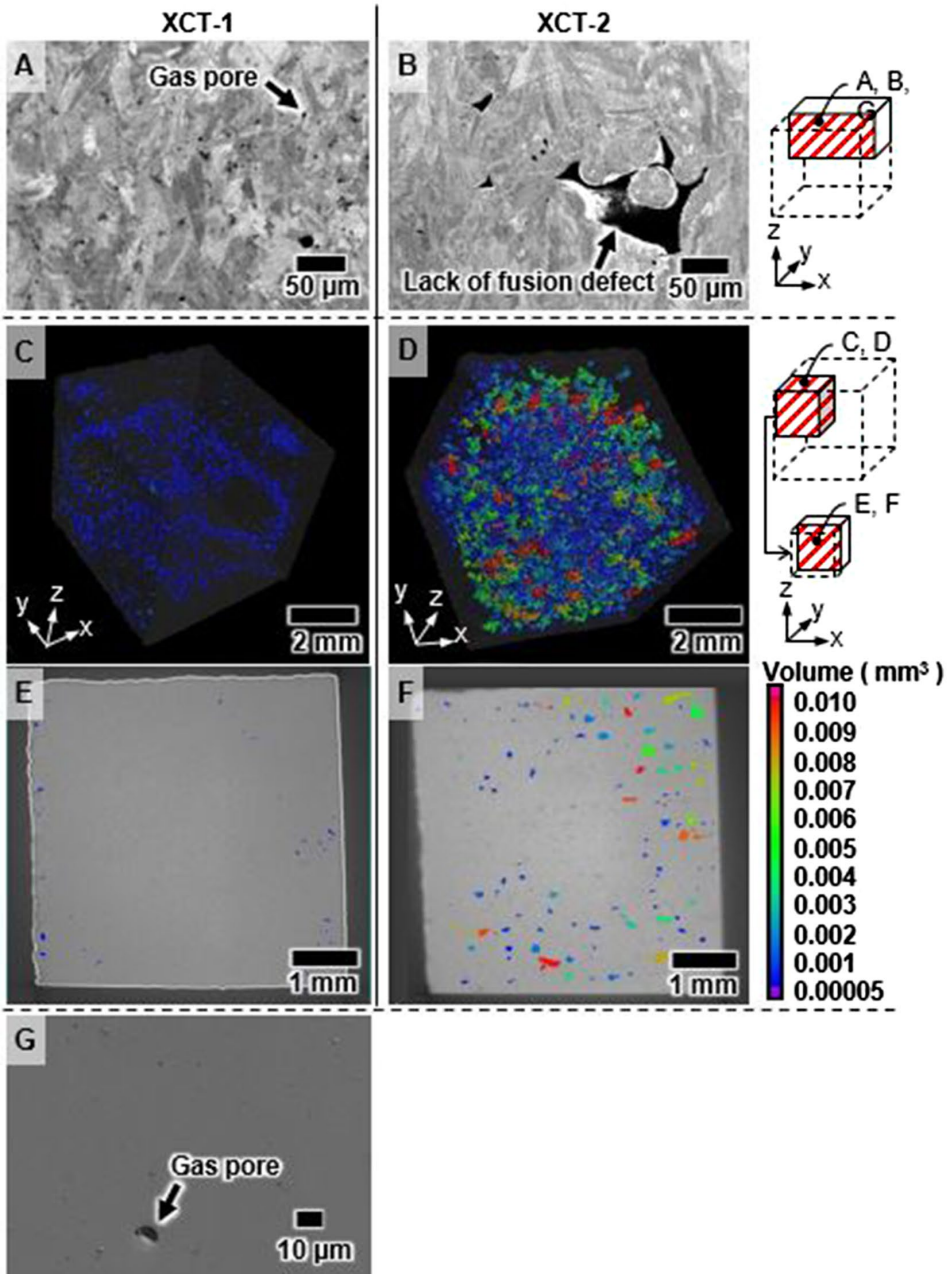
Observation of internal defects in cubic specimens—(**A**, **C**, **E**) X-CT1 images with *E* = 43.5 J/mm^3^, *D* = 100.1%; (**B**, **D**, **F**) X-CT2 images with *E* = 29.0 J/mm^3^, *D* = 98.3%. (**A**) and (**B**) depict SEM images of cubic-specimen cross-sections; (**C**) and (**D**) depict 3D XCT images; (**E**) and (**F**) depict 2D XCT images. (**G**) Image with *D* = 99.7%. Images created using PC-SEM Ver.5.1.0.11 (https://www.jeol.co.jp/) and VGStudio MAX Ver.3.4 (https://www.volumegraphics.com/jp/products/vgsm.html).

Figure 2 depicts the scanning electron microscopy (SEM) and X-ray computed tomography (XCT) images of the high- and low-density cubic specimens; these images were captured using JEOL JSM-7800F and Nikon XT H225ST, respectively. The SEM and XCT images of a full-density specimen (XCT-1) fabricated using an adequate energy density of 43.5 J/mm^3^ reveal few micropores measuring less than 10 µm in diameter (Fig. 2A, C, E). Meanwhile, the SEM and XCT images of a specimen (XCT-2) with 98% relative density fabricated at a low energy density of 29 J/mm^3^ reveal numerous large lack-of-fusion defects (Fig. 2B, D, F).

Consequently, a specimen with near-true density could be fabricated while consuming less than 500-W laser power at 2000-mm/s scan speed and energy density exceeding 35 J/mm^3^. Moreover, the structure of specimens with relative density exceeding 99.7% is characterized by the presence of micropores measuring less than ~ 10 µm in diameter (Fig. 2G). This does not significantly affect the fatigue strength of LB-PBF IN718^43,44^. Therefore, the relative density of 99.7% was set as a threshold.

**Figure 3:**
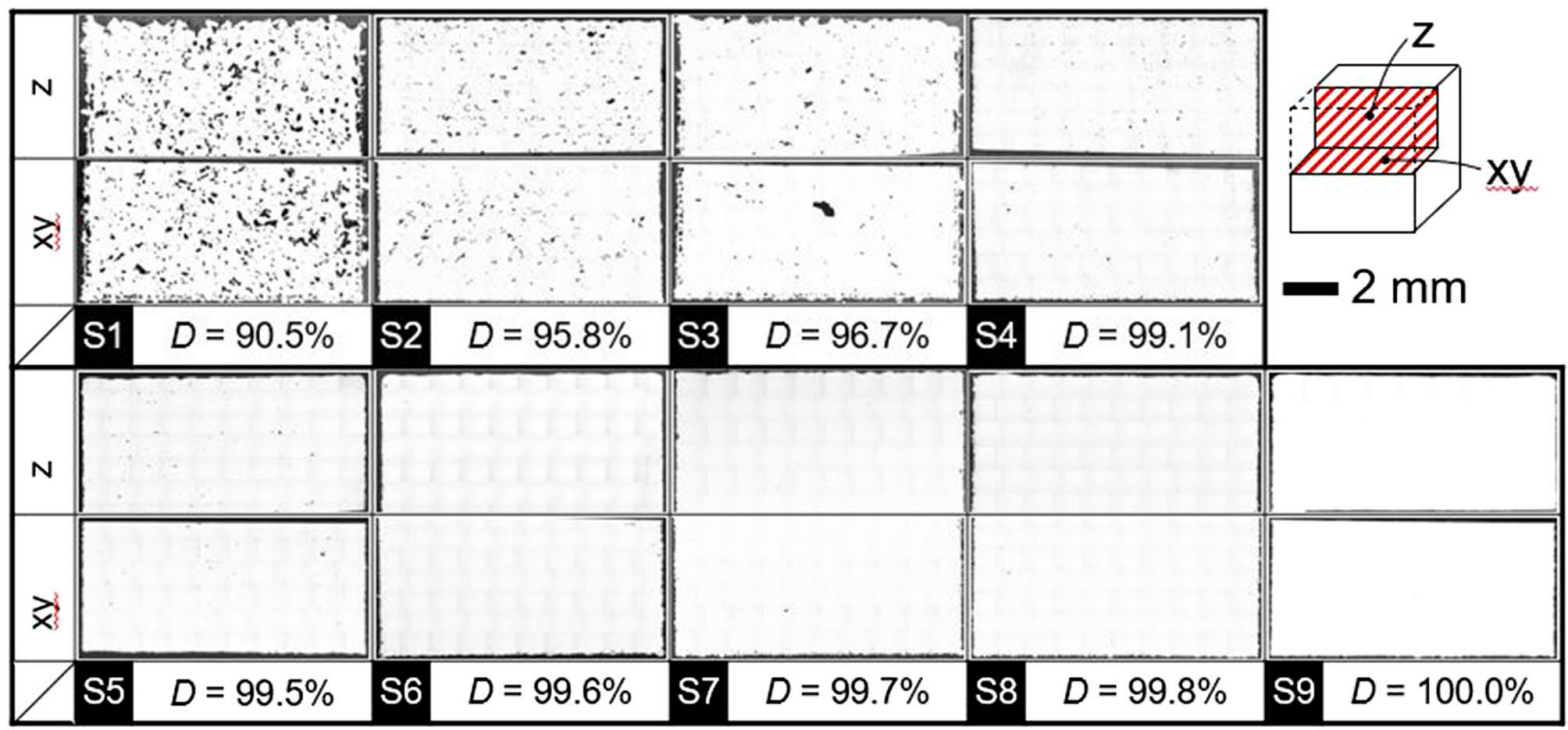
OM images of specimen (S1–S9) cross-sections. Images created using Mx Ver.7.3.0.5 (https://www.zygo.com/products/metrology-systems/metrology-software/mx-for-3d-optical-profilers).

The optical microscopy (OM) images of specimens S1–S9 (referred to in Fig. 1B, C) are presented in Fig. 3. As can be seen, very few defects exist in full-density specimens—S7, S8, and S9. Meanwhile, a few pores exist in specimens S4 with 99% relative density, whereas the lack-of-fusion defects and pores can be observed in specimens S1, S2, and S3 with low relative densities. In accordance with^20^, the lack-of-fusion defects tend to appear in materials with relative-density values below approximately 99%. Accordingly, these defects become more prominent with a decrease in the relative density of materials.

Under fabrication conditions involving the use of high laser power, micropores exist as keyhole pores or gas pores along with lack-of-fusion defects, as observed for specimens S1 and S3 in Fig. 3. Cunningham et al.^27^ elucidated the relationship between keyhole configurations and process parameters (laser power and scan speed) using the process map for the Ti–6Al–4 V alloy between laser power and scan speed with respective values of up to 500 W and 1200 mm/s. They revealed the tendency of keyhole pores to remain at the bottom of the keyhole, as reported by Martin et al.^24^, owing to the increase in keyhole depth with an increase in laser power. The analysis of the process map depicted in Fig. 1B reveals the presence of micropores without any lack-of-fusion defects in the high relative-density (> 99.7%) region. However, outside of this region, the appearance of both lack-of-fusion defects and micropores increases significantly. Accordingly, the region of low-density specimens (S1 and S3) fabricated under conditions of high power and high scan speed is marked by the presence of several lack-of-fusion defects. This confirms that the morphology of internal defects, such as lack of fusion and micropore formation, bears a strong correlation with specimen density.

### Relationship between surface texture and internal defects

**Figure 4:**
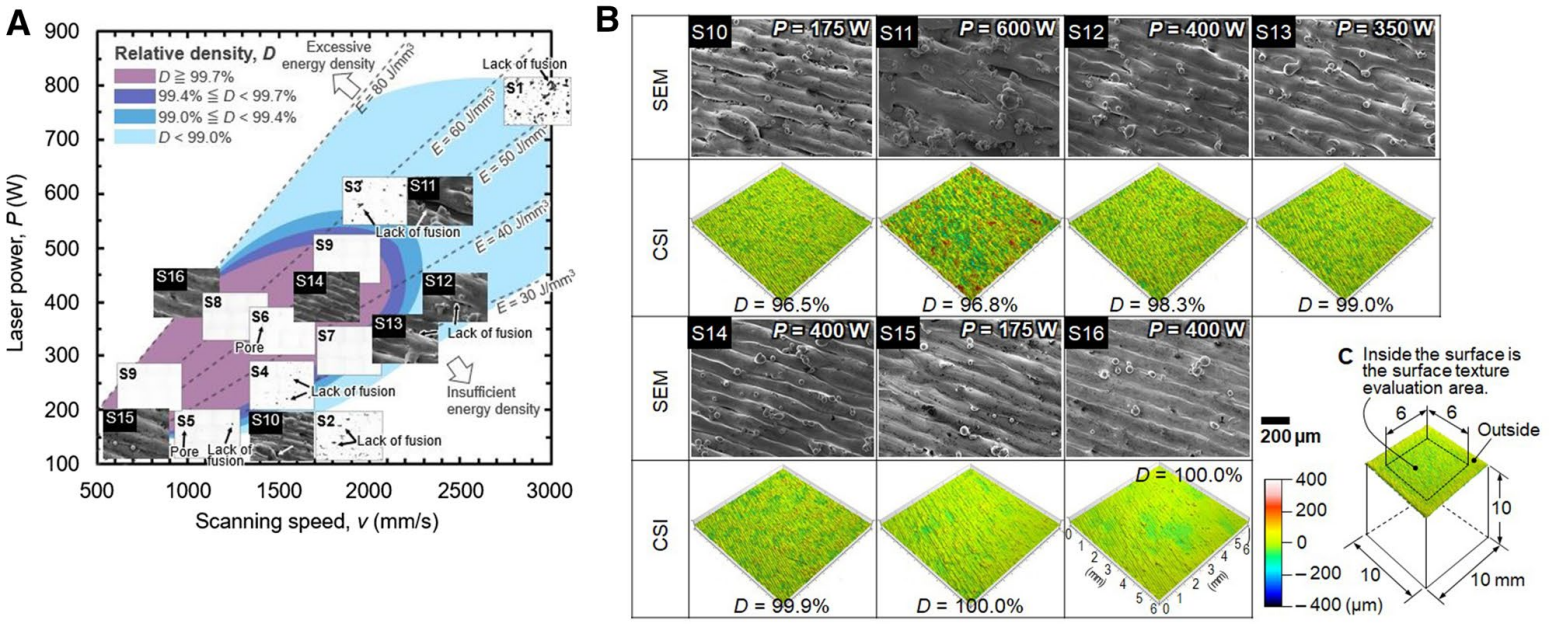
Relationship between surface texture and internal defects—(**A**) relative-density-based process map between laser power and scan speed, including surface-texture and internal-defect images; (**B**) SEM and CSI images of cubic specimen (S10–S16) surface; (**c**) surface-texture evaluation area. Images created using Microsoft Office Excel 2019 (https://www.microsoft.com/ja-jp/microsoft-365/excel), PC-SEM Ver.5.1.0.11 (https://www.jeol.co.jp/), Mx Ver.7.3.0.5 (https://www.zygo.com/products/metrology-systems/metrology-software/mx-for-3d-optical-profilers) and TalyMap Plutinum Ver.6.2.7029 (https://www.taylor-hobson.com/).

The schematic process map corresponding to Fig. 1B superimposed with the surface-texture and internal-defect of specimens S1–S16 is depicted in Fig. 4A. Reference to these figures reveals that for the high-density specimens S15 and S16, the width of the tracks (melt-pool traces) remains relatively constant, and no grooves can be observed between these tracks. As noted earlier, negligible pores exist in full-density specimens. In contrast, the said track width in low-density specimens S10, S12, and S13 remains irregular, and the surface contains several bumps and grooves. Thus, wider grooves are observed at lower laser powers, and an increase in laser power increases the width and unevenness of the tracks. Further, despite a density similar to that of S10, the surface of specimen S11 fabricated using the highest laser power (600 W) is characterized by wider tracks and grooves and greater track unevenness. This can be attributed to the large swelling (“beading-up”) of the melt pool caused by the Rayleigh–Plateau capillary instability at the high energy density^45^. Consequently, lack-of-fusion defects and pores appear in these specimens.

Thus, it could be inferred that the material density and internal defects of fabricated specimens strongly correlate with their surface texture. Therefore, the areal surface-texture parameters of all cubic specimens are quantified, and their correlation with material density is investigated to apply these parameters to monitoring and feedback systems.

Whip et al.^35^ have confirmed the adequacy of the application of the ISO 25,178–2 areal surface-texture parameters^36^ to describe the surface roughness of AM parts. In addition, Gomez et al.^38^ have reported the CSI technique to provide excellent surface-topography measurement results for metal AM surfaces with different roughness levels and slope distributions. Accordingly, CSI equipment (Zygo newview9000) was used in this study to determine the ISO25178-6 areal surface-texture parameters for the fabricated specimens. The corresponding measurements were performed by combining a 1 × zoom and 10 × objective lenses. The sampling interval was set to 0.86 µm. The field of view measured 1.37 × 1.03 mm, and the measurements of the 10 × 10 mm surface area were performed via stitching with approximately 25% overlap. The surface texture of 120 specimens was analyzed over the entire 10 × 10 mm surface. However, data pertaining to only the central 6 × 6 surface (Fig. 4C) could be extracted for analysis owing the swelling of the near-edge region of the specimen due to the wall effect^42^.

**Figure 5:**
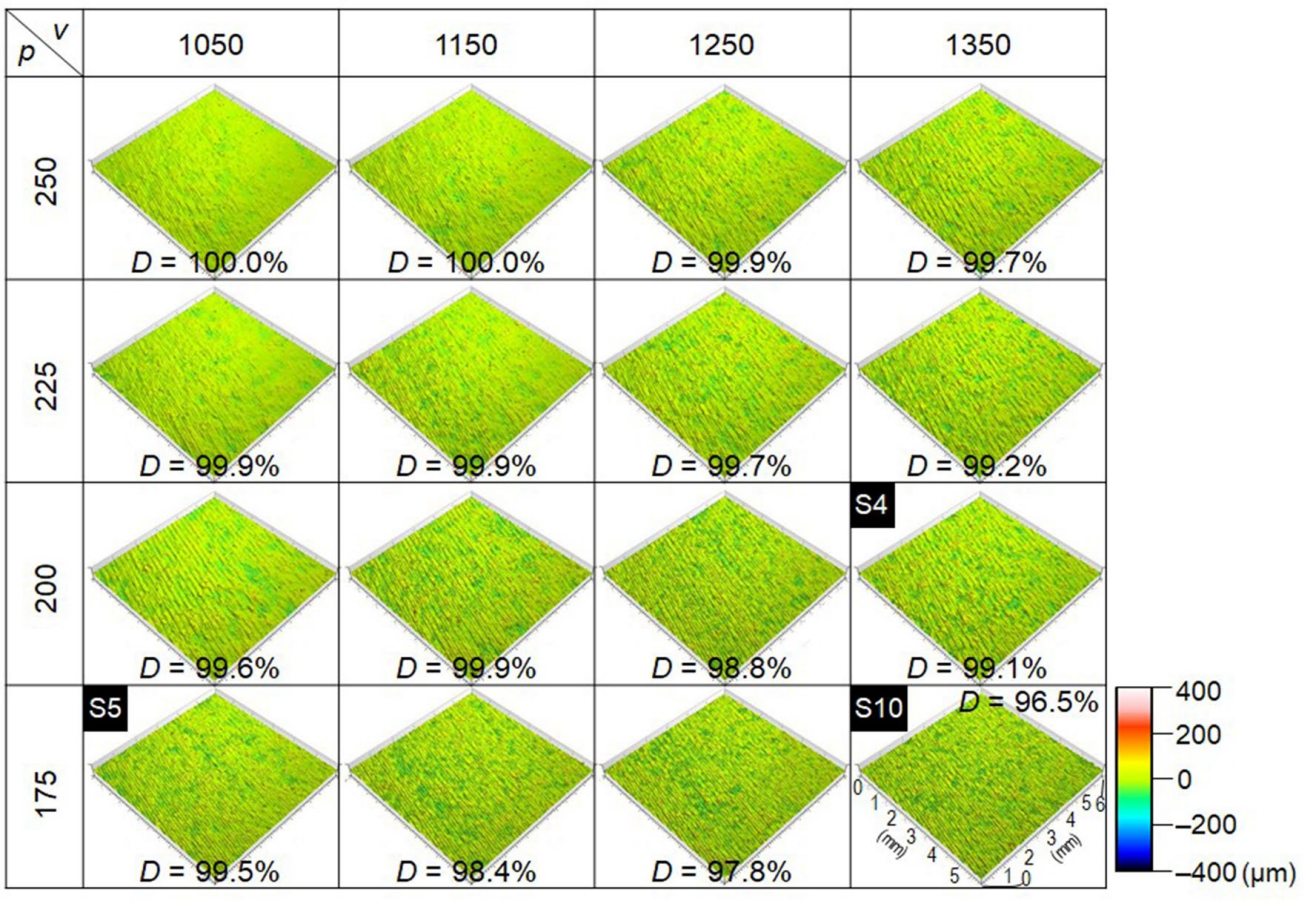
CSI images of specimens in regions of low power and low scan speed. Images created using TalyMap Plutinum Ver.6.2.7029 (https://www.taylor-hobson.com/).

The CSI and surface SEM images of specimens S10–S16 are presented in Fig. 4B. As noted above, reference to Fig. 4A reveals the surface texture of fabricated specimens to be strongly correlated with the process parameters owing to changes in the melt-pool behavior and spattering. The CSI images of specimens fabricated under low power and low scan-speed conditions are depicted in Fig. 5. At a constant laser power, the surface roughness increases with an increase in scan speed. This unevenness can be observed over the entire surface of low-density specimens. Meanwhile, at constant scan speed, the surface unevenness increases with increasing laser power owing to increased melt-pool width and instability. Compared with the surfaces shown in the SEM images depicted in Fig. 4B, the low-density specimen surfaces in Fig. 5 demonstrate more severe unevenness owing to the occurrence of grooves between tracks—i.e., lack-of-fusion defects—and spattering. In comparison, fewer defects are visible on the surfaces of specimens with intermediate densities (99–99.7%). Correspondingly, the surface texture of full-density specimens remains even owing to the formation of regular tracks and low spatter count. Thus, the CSI images can be used to evaluate the surface texture of fabricated specimens owing to their strong correspondence with SEM results. This suggests that the material density or state of internal defects of specimens can be predicted using CSI images of specimen surfaces.

### Prediction of material density and defects using areal surface-texture parameters

Previous studies^36–42^ have investigated CSI images demonstrating the influence of process parameters on the surface texture of materials. However, as discussed in the preceding sections, the relationship between process parameters and surface texture is complicated. Although prior studies^41,42^ have investigated the correlation between surface texture and material density, the correlation between surface-texture parameters and density or internal defects is yet to be quantitatively investigated.

During CSI-based surface-texture evaluation, it is difficult to perform reliable measurements for surfaces characterized by steep slopes and complicated asperity owing to insufficient laser reflection. As noted earlier, because the surfaces of the specimens reveal a complicated asperity owing to spattering and unstable track formation under certain fabrication conditions, the values of the surface-texture parameters are expected to demonstrate a large variation owing to non-measured data. The percentage of non-measured data (black area) clearly increases for specimens fabricated at a high laser power, as shown in (Supplementary Fig. 1). In addition, it is known that the density of the parts decreases at a high laser power and a high scan speed due to the occurrence of keyholing and beading up^26,27,44^. The density of the specimens is low at a laser power above 400 W, as shown in Fig. 1B. Therefore, surface texture evaluation was performed using 88 specimens fabricated at a laser power of 400 W or less.

**Figure 6:**
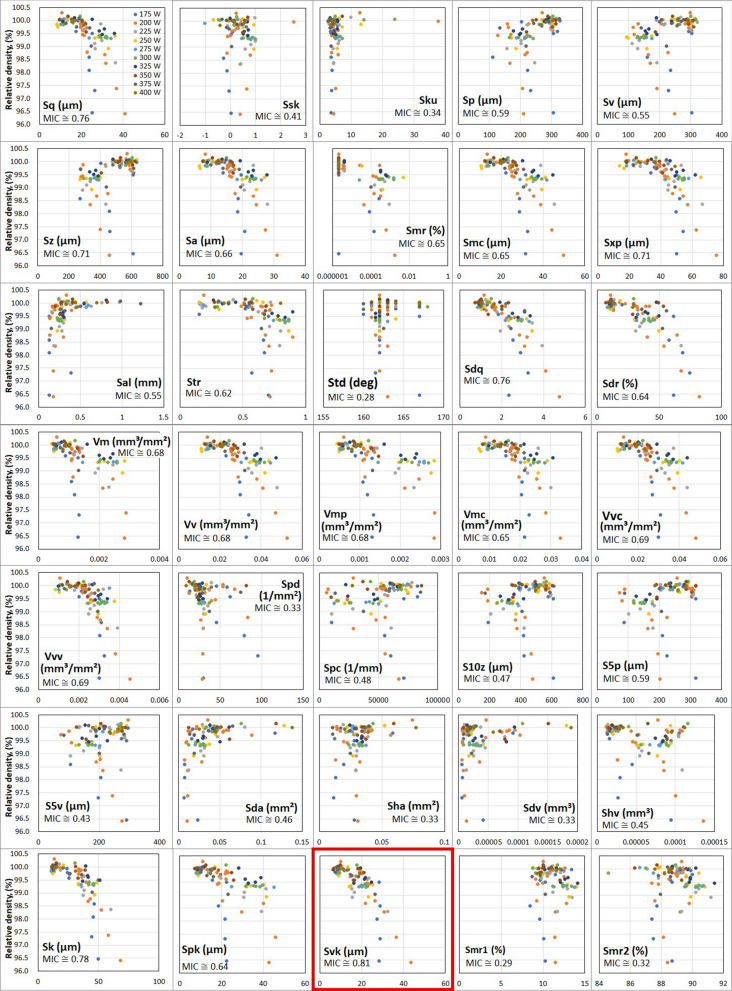
Correlation between 35 areal surface-texture parameters and relative densities of 120 cubic specimens. Images created using Microsoft Office Excel 2019 (https://www.microsoft.com/ja-jp/microsoft-365/excel).

After evaluation of the surface-texture parameters for the 88 specimens, 35 areal surface-texture parameters prescribed in the ISO 25,178–2 standard were computed using measured data. Because surface texture demonstrates a strong correlation with specimen density, a non-linear correlation analysis was performed using the maximal information coefficient (MIC)^46^. The resulting correlation between the areal surface-texture parameters and relative density of cubic specimens is depicted in Fig. 6. The corresponding values of the correlation coefficients for the topography height-distribution parameters—i.e., skewness *S*_*sk*,_ and kurtosis *S*_*ku*_—are 0.42 and 0.34, respectively. Skewness *S*_*sk*_ is the measure of the asymmetry of surface deviations around the mean plane, and kurtosis *S*_*ku*_ is the measure of peakedness or sharpness of the surface height distribution. These parameters demonstrate a weak correlation with specimen density. The corresponding values of the correlation coefficients for the height-based parameters—maximum height *S*_*z*,_ and maximum pit height *S*_*v*_—are 0.71 and 0.55, respectively. Thus, *S*_*v*_ demonstrates a weak correlation with specimen density, whereas *S*_*z*_ seems to be correlated with specimen density. Meanwhile, the reduced dale height *S*_*vk*_ reveals the highest MIC value of 0.81, whereas the core height *S*_*k*_, root-mean-square height *S*_*q*_, and root-mean-square gradient *S*_*dq*_ are 0.78, 0.76, and 0.76, respectively. In accordance with^46^, an MIC value exceeding 0.8 implies an extremely strong correlation between two variables. Thus, it is revealed that the areal surface-texture parameters *S*_*vk*_, *S*_*k*_, *S*_*q*_, and *S*_*dq*_ are strongly correlated with specimen density.

In particular, the reduced valley depth *S*_vk_ obtained by measuring the valley depth below the core roughness demonstrates a strong correlation with specimen density. It can be evaluated as the height of the right-angled triangle constructed to have an area equal to the dale area protruding out of the core surface, as defined in the ISO25178-2 standard. This implies that the gradient of the equivalent straight line in the central region of the areal material-ratio curve (i.e., *S*_vk_) increases with an increase in the difference between surface-asperity altitudes as well as the valley volume. Accordingly, the correlation between the specimen surface texture and relatively larger defects, such as lack of fusion, can be feasibly assessed using *S*_vk_.

Thus, the topography height-distribution parameters—*S*_*vk*_, *S*_*k*_, *S*_*q*_, and *S*_*dq*_—characterized by high correlation coefficients can be considered effective for evaluating the density correlation of finished parts. This inference was validated by the similarity between process maps evaluated using the relative density and the reduced dale height (*S*_*vk*_), as depicted Supplementary Fig. 2.

In summary, the proposed study reveals the existence of areal surface-texture parameters, especially *S*_*vk*_, *S*_*k*_, *S*_*q*_, and *S*_*dq*_, which are strongly correlated to the density or internal defects of LB-PBF-manufactured specimens. This finding enables the use of in-situ surface-texture monitoring of specimens to predict their density or internal defects in their structures. To this end, threshold values of the areal surface-texture parameters—*S*_*vk*_, *S*_*k*_, *S*_*q*_, or *S*_*dq*_—could be set. In addition, the in-situ monitoring of the values of these parameters can be used to realize feedback control capable of producing stable, high-quality parts via elimination of defect generation.

## Materials and methods

### Materials

In this study, gas-atomized Inconel 718 (IN718) powder (LPW Technology) was employed. The SEM image is shown in Supplementary Fig. 3. The average diameter (D50) was 29.61 µm. The flow rate (s/50 g) using a Hall flowmeter funnel and the angle of repose were 15.28 s and 28°.

### LB-PBF test bench

An LB-PBF test bench consists of a 1-kW single-mode fiber laser and a galvano laser scanner. The fiber laser wavelength was 1070 nm, and the laser diameter was 100 µm (1/e^2^). The build volume was 150 mm in diameter and 150 mm in depth. The test bench was equipped with a melt pool and surface morphology monitoring system. The specimens were fabricated in a nitrogen environment (oxygen content < 0.1 wt%).

### Coherent scanning imaging

The CSI system (ZYGO NewView™ 9000 CSI system) was applied to measure the ISO25178-6 areal surface texture parameters (Supplementary Table 1) of the specimens. The corresponding measurements were performed by combining a 1 × zoom and 10 × objective lenses. The sampling interval was set to 0.86 µm. The field of view measured was 1.37 × 1.03 mm, and the measurements of the 10 × 10 mm surface area were obtained via stitching with an overlap of approximately 25%. Examples of CSI images and measured data are shown in Supplementary Fig. 4.

## Supplementary Information

Supplementary Information.

## Data Availability

The data that support the findings of this study are available from the corresponding author on reasonable request.
