# Supplementary material for: Correlation between surface texture and internal defects in laser powder-bed fusion additive manufacturing
Source: Sci Rep. 2021 Nov 24;11:22874. doi: 10.1038/s41598-021-02240-z (PMC8613240; doi:10.1038/s41598-021-02240-z)
Supplement: Supplementary file 1 — Supplementary Information. [file 41598_2021_2240_MOESM1_ESM.docx]

**Supplementary Information**

**Correlation between surface texture and internal defects in laser powder-bed fusion additive manufacturing**

Makiko Yonehara^1,*^, Chika Kato^1,2^, Toshi-Taka Ikehsoji^1^, Koki Takeshita^2^, Hideki Kyogoku^1,*^

^1^Fundamental Technology for Next Generation Research Institute, Kindai University, 1 Takaya-Umenobe, Higashi-Hiroshima, Hiroshima 739-2116, Japan.

^2^Nikon Corporation, 6-3, Nishioi 1-chome, Shinagawa-ku, Tokyo 140-8601, Japan.

*Correspondence to: yonehara@hiro.kindai.ac.jp, kyogoku@hiro.kindai.ac.jp

The evaluation of surface area for enhancing the measurement accuracy

In this study, specimens with surfaces characterized by steep slopes and complicated asperity owing to insufficient laser reflection were excluded, as demonstrated in Supplementary Fig. 1. The surface areas of the specimen S1 fabricated at high laser power (800 W) and high scan speed (2850 mm/s) and specimen S15 fabricated at low laser power (175 W) and low scan speed (750 mm/s) are represented in Figs. S4(a) and (b), respectively. The percentages of the non-measured areas of the specimens S1 and S15 are 90.5% and 100%, respectively.

Validation of the similarity between process maps evaluated using relative density and reduced dale height (*S_vk_*)

Supplementary Fig. 2 depicts process maps evaluated using the relative density (Supplementary Fig. 2(a)), reduced dale height *S_vk_* (Supplementary Fig. 2(b)), core height *S_k_* (Supplementary Fig. 2(c)), root mean square height *S_q_* (Supplementary Fig. 2(d)), and root mean square gradient *S_dq_* (Supplementary Fig. 2(e)), specifically for the low-power and low-scan-speed region. As can be seen, the two process maps are nearly identical. This implies that *S_vk_* is highly effective in evaluating the density of and defects within finished parts. This result is indicative of the possible fabrication of high-density finished parts by exclusively monitoring *S_vk_* without the need to evaluate the density of finished parts. Finally, the in-situ monitoring of parameters *S_vk_*, *S_k_*, *S_q_*, and *S_dq_* enables their application as feedback control variables to ensure high quality of parts manufactured using the LB-PBF process.

**Figures S1 – S4**

A


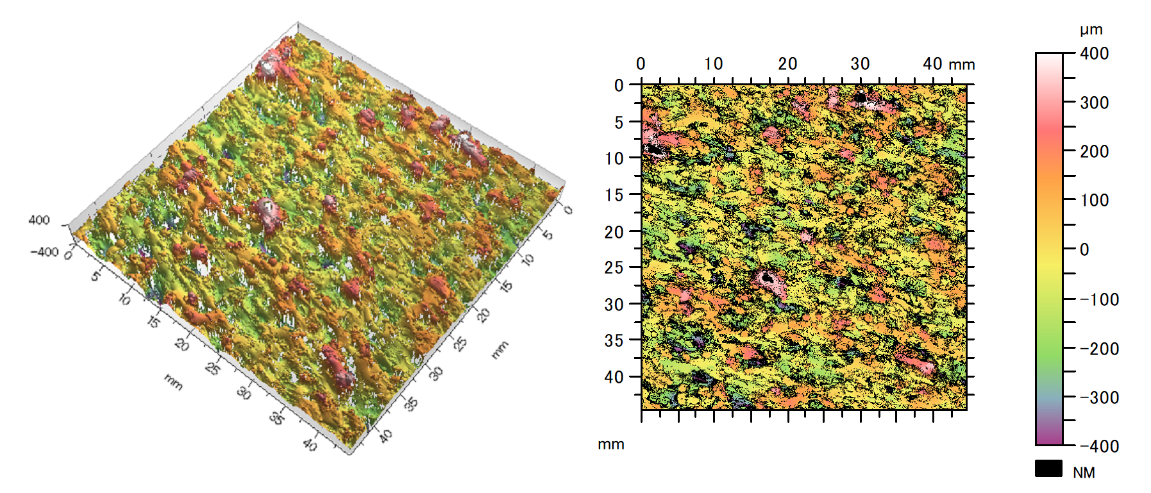


B


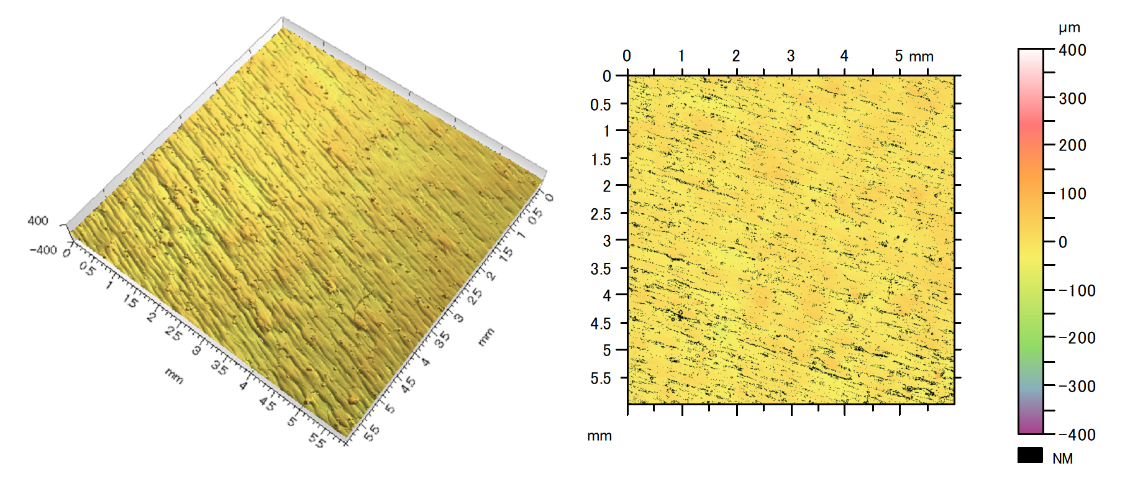


**Supplementary Fig. 1**. Examples of specimens with surfaces characterized by steep slopes and complicated asperity owing to insufficient laser reflection (black area). (**a**) S1 specimen fabricated at high laser power and high scan speed. (**b**) S15 specimen fabricated at low laser power and low scan speed. Images created using TalyMap Plutinum Ver.6.2.7029 (https://www.taylor-hobson.com/).


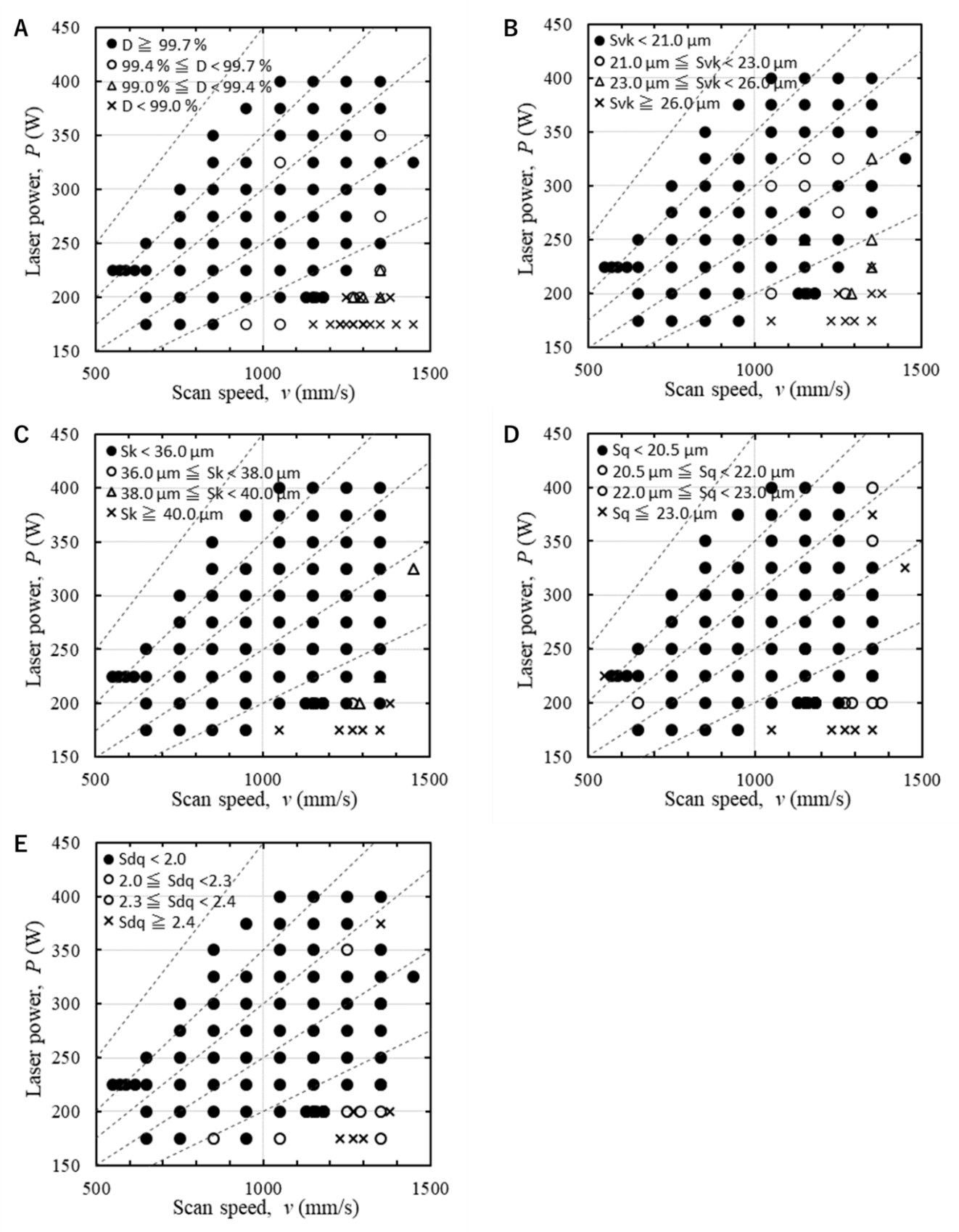


**Supplementary Fig. 2.** Comparison between process maps evaluated using (**a**) relative density, (**b**) reduced dale height (*S_vk_*), (**c**) core height (*S_k_*), (**d**) root mean square height (*S_q_*), and (**e**) root mean square gradient (*S_dq_*) in low-power and low-scan-speed region. Images created using Microsoft Office Excel 2019 (<https://www.microsoft.com/ja-jp/microsoft-365/excel>).


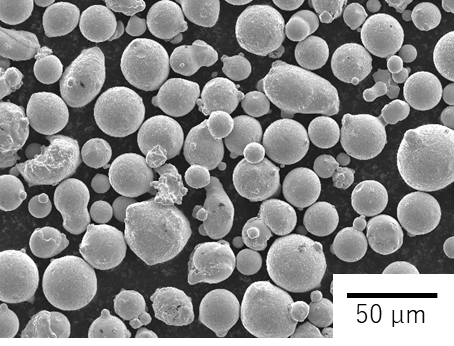


**Supplementary Fig. 3**. SEM image of IN718 powder. Images created using PC-SEM Ver.5.1.0.11 (https://www.jeol.co.jp/).


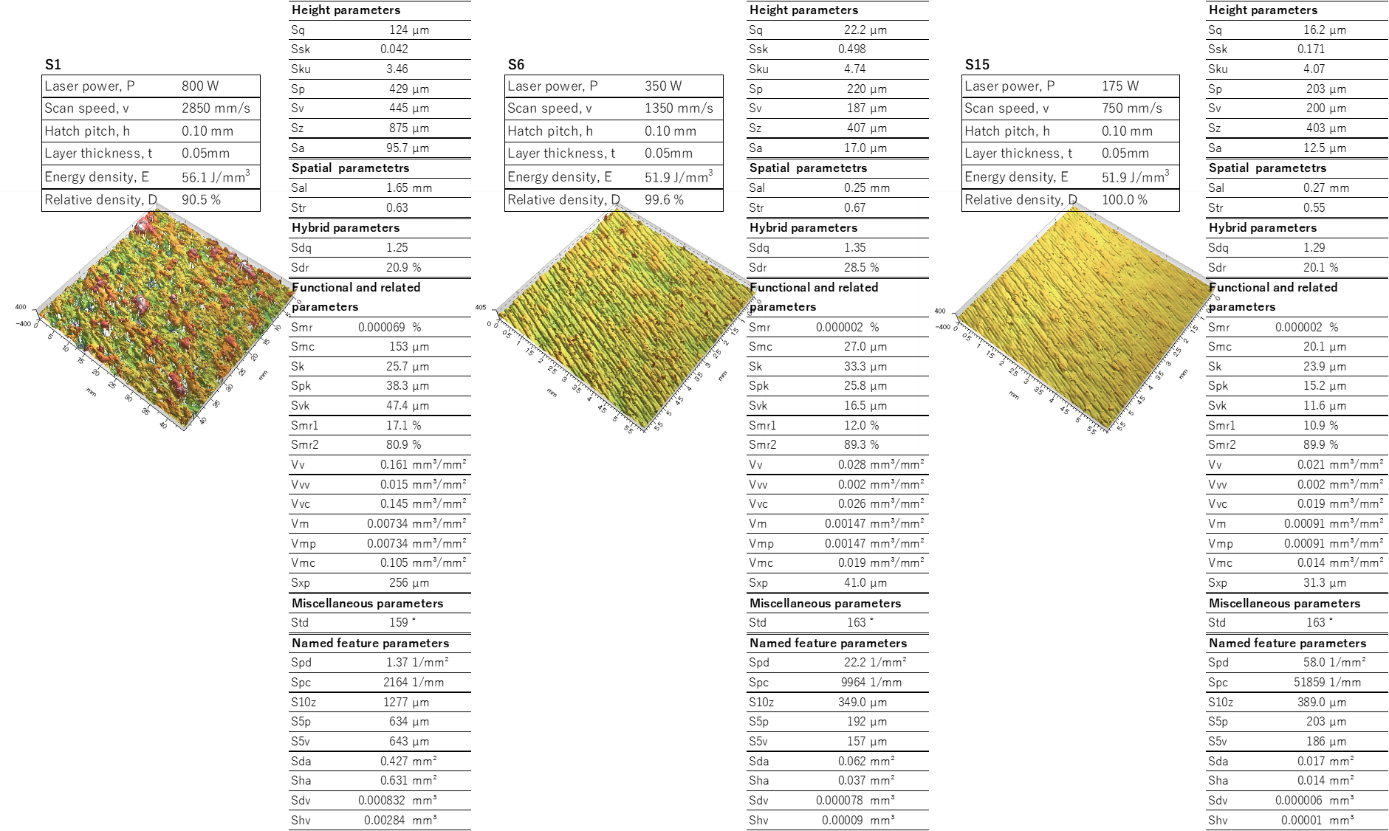


**Supplementary Fig. 4**. Examples of SCI images measured using the coherent scanner system. Images created using TalyMap Plutinum Ver.6.2.7029 (https://www.taylor-hobson.com/).

Supplementary Table 1.

Surface-texture parameters defined by ISO 25178-6

| Height parameters | | |  |
| --- | --- | --- | --- |
| 1 | S_q_ | µm | Root-mean-square height |
| 2 | S_sk_ |  | Skewness |
| 3 | S_ku_ |  | Kurtosis |
| 4 | S_p_ | µm | Maximum peak height |
| 5 | S_v_ | µm | Maximum pit height |
| 6 | S_z_ | µm | Maximum height |
| 7 | S_a_ | µm | Arithmetical mean height |
| Spatial parameters | | |  |
| 8 | S_al_ | mm | Autocorrelation length |
| 9 | S_tr_ |  | Texture aspect ratio |
| Hybrid parameters | | |  |
| 10 | S_dq_ |  | Root-mean-square gradient |
| 11 | S_dr_ | % | Developed interfacial area ratio |
| Functions and related parameters | | | |
| 12 | S_mr_ | % | Areal material ratio |
| 13 | S_mc_ | µm | Inverse areal material ratio |
| 14 | S_k_ | µm | Core height |
| 15 | S_pk_ | µm | Reduced peak height |
| 16 | S_vk_ | µm | Reduced dale height |
| 17 | S_mr1_ | % | Material ratio |
| 18 | S_mr2_ | % | Material ratio |
| 19 | V_v_ | mm³/mm² | Void volume |
| 20 | V_vv_ | mm³/mm² | Dale void volume |
| 21 | V_vc_ | mm³/mm² | Core void volume |
| 22 | V_m_ | mm³/mm² | Material volume |
| 23 | V_mp_ | mm³/mm² | Peak material volume |
| 24 | V_mc_ | mm³/mm² | Core material volume |
| 25 | S_xp_ | µm | Peak extreme height |
| Miscellaneous parameters | | | |
| 26 | S_td_ | ° | Texture direction |
| Named feature parameters | | | |
| 27 | S_pd_ | 1/mm² | Density of peak |
| 28 | S_pc_ | 1/mm | Arithmetical mean peak curvature |
| 29 | S_10z_ | µm | Ten-point height of surface |
| 30 | S_5p_ | µm | Five-point peak height |
| 31 | S_5v_ | µm | Five-point pit height |
| 32 | S_da_ | mm² | Mean dale area |
| 33 | S_ha_ | mm² | Mean hill area |
| 34 | S_dv_ | mm³ | Mean dale volume |
| 35 | S_hv_ | mm³ | Mean hill volume |
